# *Notes from the Field:* Acute Nonviral Hepatitis Linked to a Brand of Alkaline Bottled Water — Clark County, Nevada and California, 2020

**DOI:** 10.15585/mmwr.mm7046a6

**Published:** 2021-11-19

**Authors:** Jeanne C. Ruff, Ying Zhang, David P. Bui, Colin Therriault, Daniel Nogee, Stephen L. Guthery, Johnni Daniel

**Affiliations:** ^1^Division of Scientific Education and Professional Development, CDC; ^2^Epidemic Intelligence Service, CDC; ^3^Southern Nevada Health District, Las Vegas, Nevada; ^4^National Center for Environmental Health, CDC; ^5^Emory University School of Medicine, Atlanta, Georgia; ^6^Intermountain Primary Children’s Hospital, Salt Lake City, Utah; ^7^Department of Pediatrics, University of Utah, Salt Lake City, Utah.

During November 10–December 3, 2020, five previously healthy children aged 7 months–5 years in Clark County, Nevada, were hospitalized with lethargy and hypoglycemia after vomiting for several days. Clinical findings satisfied criteria for acute liver failure (*1*), and the children were transferred to a tertiary care children’s hospital for potential liver transplantation. No etiology was identified despite extensive medical evaluations. The Southern Nevada Health District (SNHD) was notified of this unusual cluster, and staff members reviewed medical records and interviewed parents to identify common exposures. Multiple household members from two households reported vomiting within the same time frame as the children, and one adult household member had been hospitalized with unexplained liver abnormalities several months earlier. Further investigation revealed that all patients and symptomatic household members consumed “Re^2^al Alkalized Water” brand bottled water before illness onset; no other shared exposures were reported.

On March 13, 2021, SNHD, the Nevada Department of Health and Human Services, CDC, and the Food and Drug Administration (FDA) launched a public health investigation to assess the extent of the outbreak, identify the substance or agent causing the illnesses, and mitigate public risk. Possible cases were mainly identified through self-report and clinician report. Local health jurisdictions interviewed patients and reviewed medical records. A probable case of liver illness associated with Re^2^al Water consumption included the following criteria: 1) new onset hepatitis of unknown etiology (HUE)* with symptom onset on or after August 1, 2020, after Re^2^al Water consumption ≤30 days before illness onset; 2) documentation of a negative viral hepatitis panel and hepatic imaging that did not reveal a cause, and 3) no documentation of another cause. Because the causative agent had not been identified, no confirmed case definition was proposed. A suspected case definition was established to allow for variation in clinical workup; a suspected case met only the first and third probable case criteria. Re^2^al Water offered a variety of products including 5-gallon home delivery available regionally and smaller bottles available nationwide in grocery stores and through online vendors. Therefore, case-finding was not limited by geographic region. This activity was reviewed by CDC and was conducted consistent with applicable federal law and CDC policy.^†^

At least four states, Nevada, California, Arizona, and Utah, conducted case-finding efforts. Eighteen probable and four suspected cases were identified in Nevada and three probable cases in California. This report describes the 21 probable cases.

Most patients had illness onset in November 2020 (Figure). Apart from the five children, all were aged >30 years at illness onset. Frequently reported symptoms included fatigue (19; 90%), vomiting (18; 86%), decreased appetite (18; 86%), dizziness or vertigo (13; 62%), and unintentional weight loss (11; 52%). All persons with probable cases required hospitalization; 18 (86%) required intensive care unit admission. Laboratory liver function indicators were markedly abnormal.^§^ Liver transplantation was initially anticipated for multiple patients (*2*); however, all patients considered for transplant recovered without transplant. One patient, a woman aged in her 60s with underlying medical conditions, died of HUE–related complications in November 2020. All patients with probable cases consumed water from the 5-gallon size product before illness onset.

**FIGURE:**
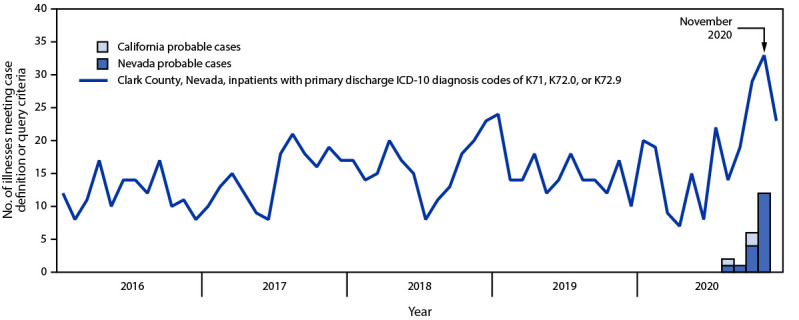
Probable cases of acute nonviral hepatitis linked to alkaline bottled water among residents of Nevada and California, by month of illness onset during August–December 2020* and trends in monthly inpatient diagnoses of toxic liver disease or acute hepatic failure, unspecified — Clark County, Nevada,^†^^,^^§^ 2016–2020 **Abbreviation:** ICD-10 = *International Classification of Diseases, Tenth Revision*. * Eighteen probable cases were identified in Nevada and three in California. Probable cases met the following criteria: new onset hepatitis of unknown etiology with symptom onset on or after August 1, 2020, after “Re^2^al Alkalized Water” brand bottled water consumption ≤30 days before illness onset; documentation of a negative viral hepatitis panel and hepatic imaging that did not reveal a cause; and no documentation of another cause. ^†^ There is no ICD-10 code specific for the liver illness seen in this outbreak. However, a query consisting of a combination of existing ICD-10 codes detected 14 of Clark County’s 18 probable cases in Clark County, Nevada, nonfederal acute care hospital inpatient billing data. It has not been determined whether other illnesses identified with this query meet the probable case definition criteria. ^§^ Query terms were as follows: primary discharge ICD-10 diagnosis code of toxic liver disease (K71) or hepatic failure, not elsewhere classified (K72.0 or K72.9) and no discharge diagnosis of chronic hepatic failure (K72.1), acetaminophen poisoning or adverse effect (T39.1X1, T39.1X2, T39.1X3, T39.1X4, and T39.1X5), autoimmune hepatitis (K75.4), primary biliary cirrhosis (K74.3), primary sclerosing cholangitis (K83.01), Wilson’s disease (E83.01), hemochromatosis (E83.11), viral hepatitis (B15, B16, B17, B18, and B19), Reye’s syndrome (G93.7), alcoholic liver disease (K70), alcohol abuse (F10), or chronic hepatitis, not elsewhere classified (K73).

Clinical findings were consistent with possible toxic exposure. Alkaline water has not previously been associated with hepatotoxicity, and evidence does not indicate involvement of other brands. As is common in toxicological outbreak investigations, the substance likely to have caused the illnesses has not been identified. It is unclear whether only the 5-gallon size product was affected or whether those who consumed water from the 5-gallon size product consumed more Re^2^al Water than people who consumed water from the other sizes. Given this uncertainty, the manufacturer, Real Water, Inc., began a voluntary recall of all products on March 17, 2021 (*3*,*4*). On June 1, 2021, the company agreed to cease operations until requirements of a consent decree are met (*5*).

Most jurisdictions do not have an established surveillance system for HUE, nor is there an *International Classification of Diseases, Tenth Revision* (ICD-10) code for HUE. However, a query of ICD-10 codes in nonfederal acute care hospital inpatient billing data (*6*) in Clark County, Nevada showed an increase in patients discharged with primary diagnoses of “toxic liver disease” or “hepatic failure, not elsewhere classified” during October and November 2020, after codes for known causes of liver injury were excluded (Figure). The ability to identify probable cases with this query was tested by searching query output for the 18 previously identified probable cases from Clark County, 14 of which were detected. It has not been determined whether other illnesses identified with this query meet the probable case definition criteria. This investigation illustrates the importance of reporting unusual illnesses to public health authorities.

## Footnotes

* Required laboratory parameters were serum aspartate aminotransferase (AST) or alanine transaminase (ALT) ≥4 times the upper limit of normal (ULN), or alkaline phosphatase ≥2 times the ULN, or an elevated AST or ALT and total bilirubin ≥2 times the ULN.

† 45 C.F.R. part 46, 21 C.F.R. part 56; 42 U.S.C. Sect. 241(d); 5 U.S.C. Sect. 552a; 44 U.S.C. Sect. 3501 et seq.

§ The ULN for liver function indicators vary by age, sex, and laboratory. Therefore, values are expressed in terms of the measured value divided by the ULN that was listed for each patient. Peak aspartate transaminase levels were 8.5–359.9 times the ULN (median = 68.3 times ULN). Alanine aminotransferase levels were 3.1–77.4 times the ULN (median = 27.0 times ULN). Alkaline phosphatase levels were 0.8–2.7 times the ULN (median = 1.4 times ULN). Total bilirubin levels were 1.1–18.2 times the ULN (median = 5.5 times ULN). International normalized ratio (INR) values (available for 16 of 21 probable cases) were 1.73–11.39 (median = 5.24; normal value for patients not on anticoagulation therapy = approximately 1). For two patients with probable cases, INR values were above the laboratory’s reporting limit, and were reported as >8.0 and >8.7. INR values were not available for three patients with probable HUE.

## References

[1] Squires RH Jr, Shneider BL, Bucuvalas J, Acute liver failure in children: the first 348 patients in the pediatric acute liver failure study group. J Pediatr 2006;148:652–8.e2. 10.1016/j.jpeds.2005.12.051 16737880 PMC2662127

[2] Berk C, Ammar T, Lee BT. Acute liver failure due to manufactured alkaline water: a case series of “real water”-induced liver injury. Am J Gastroenterol 2021. Epub July 5, 2021. 34223827 10.14309/ajg.0000000000001353

[3] Food and Drug Administration. Event details. Silver Spring, MD: US Department of Health and Human Services, Food and Drug Administration; 2021. Accessed May 21, 2021. https://www.accessdata.fda.gov/scripts/ires/index.cfm?Event=87552

[4] Food and Drug Administration. Real Water, Inc., issues precautionary recall of all sizes of Real Water brand drinking water due to a possible health risk. Silver Spring, MD: US Department of Health and Human Services, Food and Drug Administration; 2021. https://www.fda.gov/safety/recalls-market-withdrawals-safety-alerts/real-water-inc-issues-precautionary-recall-all-sizes-real-water-brand-drinking-water-due-possible

[5] Food and Drug Administration. Nevada-based bottled water manufacturer agrees to stop production for failure to comply with manufacturing requirements. Silver Spring, MD: US Department of Health and Human Services, Food and Drug Administration; 2021. https://www.fda.gov/news-events/press-announcements/nevada-based-bottled-water-manufacturer-agrees-stop-production-failure-comply-manufacturing

[6] Center for Health Information Analysis for Nevada. About us. Las Vegas, NV: University of Nevada, Las Vegas; 2021. https://chiaunlv.com/AboutUs/AboutUs.php

